# Collagen-Based Biomaterials for Tissue Engineering Applications

**DOI:** 10.3390/ma3031863

**Published:** 2010-03-16

**Authors:** Rémi Parenteau-Bareil, Robert Gauvin, François Berthod

**Affiliations:** 1 Laboratoire d’Organogénèse Expérimentale (LOEX), Centre de recherche FRSQ du CHA universitaire de Québec, Hôpital du Saint-Sacrement, Québec, QC, G1S 4L8 Canada; E-Mails: remi.parenteau.1@ulaval.ca (R.P.B.); robert.gauvin.1@ulaval.ca (R.G.); 2 Département de chirurgie, Faculté de médecine, Université Laval, Québec, QC, G1V 0A6 Canada

**Keywords:** collagen, biomaterial, soft tissue

## Abstract

Collagen is the most widely distributed class of proteins in the human body. The use of collagen-based biomaterials in the field of tissue engineering applications has been intensively growing over the past decades. Multiple cross-linking methods were investigated and different combinations with other biopolymers were explored in order to improve tissue function. Collagen possesses a major advantage in being biodegradable, biocompatible, easily available and highly versatile. However, since collagen is a protein, it remains difficult to sterilize without alterations to its structure. This review presents a comprehensive overview of the various applications of collagen-based biomaterials developed for tissue engineering, aimed at providing a functional material for use in regenerative medicine from the laboratory bench to the patient bedside.

## 1. Introduction 

During the past decade, numerous innovations occurred in the field of collagen-based biomaterials. From injectable collagen matrices to bone regeneration scaffolds, production and cross-linking methods have evolved and improved. Collagen is now widely used in both research environments and medical applications. A brief introduction of the collagen molecule will be presented, followed by some technical explanation covering collagen scaffold production methods. Ultimately, the recent advances that have been developed in collagen-based tissue-engineering biomaterials will be discussed. 

## 2. The Collagen Molecule

### 2.1. Distribution, biosynthesis and molecular structure

The presence of collagen in all connective tissue makes it one the most studied biomolecules of the extracellular matrix (ECM). This fibrous protein species is the major component of skin and bone and represents approximately 25% of the total dry weight of mammals [1]. To this day, 29 distinct collagen types have been characterized and all display a typical triple helix structure. Collagen types I, II, III, V and XI are known to form collagen fibers. Collagen molecules are comprised of three α chains that assemble together due to their molecular structure. Every α chain is composed of more than a thousand amino acids based on the sequence -Gly-X-Y-. The presence of glycine is essential at every third amino acid position in order to allow for a tight packaging of the three α chains in the tropocollagen molecule and the X and Y positions are mostly filled by proline and 4-hydroxyproline [2,3].

There are approximately twenty-five different α chain conformations, each produced by their unique gene. The combination of these chains, in sets of three, assembles to form the twenty-nine different types of collagen currently known. The most common are briefly described in Table 1. Although many types of collagen have been described, only a few types are used to produce collagen-based biomaterials. Type I collagen is currently the gold standard in the field of tissue-engineering.

**Figure 1:**
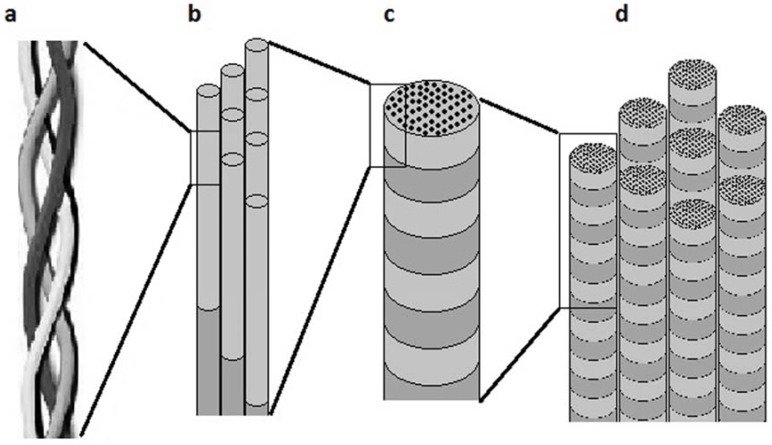
(a) Schematization of a collagen α chain triple helix segment. (b) Assembled tropocollagen molecules. (c) Collagen fibril ranging from 10 to 300 nm in diameter. (d) Aggregated collagen fibrils forming a collagen fiber with a diameter ranging from 0.5 to 3 μm.

The fibroblast is responsible for the majority of the collagen production in connective tissue. Collagen pro-α chain is synthesized from a unique mRNA within the rough endoplasmic reticulum and is then transferred to the Golgi apparatus of the cell. During this transfer, some prolines and lysines residues are hydroxylated by the lysyl oxydase enzyme. Specific lysines are glycosylated and then pro-α chains self-assemble into procollagen prior to their encapsulation in excretory vesicles. Following their passage through the plasma membrane, the propeptides are cleaved outside the cell to allow for the auto-polymerisation by telopeptides. This step marks the initiation of tropocollagen self-assembly into 10 to 300 nm sized fibril and the agglomeration of fibril into 0.5 to 3 μm collagen fibers, see Figure 1 [1]. Fibril-forming collagens are the most commonly used in the production of collagen-based biomaterials.

**Table 1:** Collagen types, forms and distribution. Modified from [1].

	Type	Molecular formula	Polymerized form	Tissue distribution
Fibril-Forming (fibrillar)	I	[α1(I)]_2_α2(I)	fibril	bone, skin, tendons, ligaments, cornea (represent 90% of total collagen of the human body)
	II	[α1(II)]_3_	fibril	cartilage, intervertebrate disc, notochord, vitreous humor in the eye
	III	[α1(III)]_3_	fibril	skin, blood vessels
	V	[α1(V)]_2_α2(V) and α1(V)α2(V)α3(V)	fibril (assemble with type I)	*idem* as type I
	XI	α1(XI)α2(XI)α3(XI)	fibril (assemble with type II)	*idem* as type II
Fibril-associated	IX	α1(IX)α2(IX)α3(IX)	lateral association with type II fibril	cartilage
	XII	[α1(XII)]_3_	lateral association with type I fibril	tendons, ligaments
Network-forming	IV	[α1(IV)]_2_α2(IV)	Sheet-like network	basal lamina
	VII	[α1(VII)]_3_	anchoring fibrils	beneath stratified squamous epithelia

### 2.2. Immunogenicity and biocompatibility

The use of biological material for medical applications requires making a distinction between immunogenicity and antigenicity. Immunogenicity is about triggering an immune response while antigenicity refers to the interaction between the antibodies and the antigenic determinants or epitopes [4]. An immune response against collagen mainly targets epitopes in the telopeptide region at each end of the tropocollagen molecule [5,6,7]. However, the conformation of the helical part and the amino acid sequence on the surface of the polymerized collagen fibril, also influence the immunologic profile of the collagen molecule [8,9,10,11,12]. Thus, the difference of immunogenicity between polymerized collagen and their smaller counterpart lies on the accessibility of the antigenic determinants that decrease during the polymerisation process [13,14].

Type I collagen is a suitable material for implantation since only a small amount of people possess humoral immunity against it and a simple serologic test can verify if a patient is susceptible to an allergic reaction in response to this collagen-based biomaterial [15,16]. 

It is important to mention that these facts about collagen immunogenicity are also applicable to collagen molecules comprised of an acellular ECM and that most adverse immune responses that have been encountered with an acellular scaffold are not necessarily originating from the collagen molecule itself. Incomplete decellularization resulting in residual oligosaccharide α-Gal or DNA is most often the cause of acellular ECM rejection or acute immune responses [17].

### 2.3. Collagen origin and variability

Collagen can be extracted from various sources considering that it is one of the most abundant proteins on earth. It can be extracted from almost every living animal, even including alligators [18] and kangaroos [19]. Nonetheless, common sources of collagen for tissue engineering applications include bovine skin and tendons, porcine skin and rat tail among others. Marine life forms are also a considerable source of collagen, which can be extracted from sponges [20,21], fish [22] and jellyfish [23]. These collagens are widely used in the industry, but less for research and clinical usage. All these collagen sources are worth investigating considering that collagen properties differ from one animal to another [24]. However, the company Fibrogen® distributes recombinant human collagen since 2004 that is potentially less immunogenic than animal sources but more importantly is identical in composition for different production lots and appears to be the future of collagen scaffolds [25]. Collagen can also be used in biomedical applications as a decellularized ECM serving as a scaffolding material for tissue regeneration. Although extractible from many different sources, the diversity of acellular collagen scaffolds are quite restraint due to immunological, physical scaffold size and availability aspects. Thus, acellular ECM are typically produced from human or porcine dermis or from swine intestine or bladder submucosa (SIS or BSM) [26].

### 2.4. Biodegradability and collagenases

Biodegradability is a valuable aspect for most collagen-based biomaterials. Collagen biocompatibility and possible degradation by human collagenases are responsible for the widespread use of this material in many biomedical applications. On the other hand, the rate of the degradation process often needs to be regulated using diverse methods such as crosslinking techniques [13,14] or a structural modification agent like Epigallocatechin-3-gallate (EGCG) [27]. Therefore, biodegradation of collagen-based biomaterials for applications such as tissue engineering could potentially lead to the restoration of tissue structure and functionality [28]. In addition, the degradation product of collagen type I to III have also been shown to induce a chemotactic attraction of human fibroblasts [29]. 

Collagenases such as matrix metalloproteinase (MMP) are responsible for most collagen degradation *in vivo*. It is also important to know that all collagenases have a different rate of collagen hydrolysis. Mammalian collagenases such as MMP-1, MMP-2, MMP-8, MMP-13 and MMP-14 have the capacity to hydrolyze collagen type I to III [30,31,32,33,34,35], while some other like MMP-3 and MMP-9 bind to type I collagen but do not degrade the native tropocollagen molecule [36,37,38]. The collagenolytic activity of all these MMP rely on three principles: the ability to bind collagen molecules, the ability to unwind the three α chains and the ability to cleave each strand of the triple helix [39]. These parameters are also of concern when it comes to bacterial collagenases or non-specific proteolytic enzymes such as *C. histolyticum* collagenase or trypsin.

### 2.5. Collagen and cellular interactions

Collagen is a key structural element of vertebrate evolution. The path that led to complex life form, like human being, relies on three types of interactions. In 1985, Ruoslahti *et al.* stated that relation between cells and ECM are based on self-aggregation of matrix molecules, on interaction of these aggregated molecules with one another and finally on their affinity toward cell surface to allow cells binding to the ECM as well as proliferation [40]. Cell-matrix interactions imply mostly interaction of cells with collagen, directly or indirectly. Direct cell-collagen interactions imply cell receptors that recognize specific peptide sequence within collagen molecules. These receptors are divided into four groups. The receptors of the first group, like glycoprotein VI, recognize peptide sequence containing GPO motif (Gly-Pro-Hyp) [41]. The second group is composed of collagen binding receptor members of integrin family and discoidin domain receptor 1 and 2 (DDR1 and DDR2). All these receptors bind to different specific motifs often including a GFO (Gly-Phe-Hyp) sequence [42,43,44]. The third group of collagen binding receptor are integrin-types that recognize cryptic motifs within the collagen molecule [45]. Finally the other cell receptors that directly bind collagen have affinity for the non-collagenous domain of the molecule. The two last groups of collagen binding receptors normally imply other cell-matrix interactions via indirect cell-collagen interactions to achieve stable adhesion of cell to the ECM. One of the key molecules of indirect cell-collagen interactions is fibronectin, on which the integrin recognized sequence RGD (Arg-Gly-Asp) was first identified [46,47]. A lot of proteins containing RGD or similar motifs recognized by integrin also bind to collagen, thus allowing indirect cell-collagen interactions. Proteins like decorin and laminin can bind either collagen and integrin promoting cell adhesion and proliferation [48]. Those facts about collagen receptors and collagen binding molecule are of important concern for the choice of collagen or ECM source to produce collagen-based biomaterials. This is why the treatments used to extract collagen, to decellularized ECM or to sterilized biomaterials are of outmost importance. Furthermore, the molecular architecture of collagen and other associated proteins in biomaterials are crucial for seeded cell adhesion, migration and in some case differentiation.

## 3. Collagen-Based Biomaterials

### 3.1. Types of collagen-based biomaterials

Collagen-based biomaterials can originate from two fundamental techniques. The first one is a decellularized collagen matrix preserving the original tissue shape and ECM structure, while the other relies on extraction, purification and polymerization of collagen and its diverse components to form a functional scaffold. Both techniques can be submitted to various cross-linking methods and protocols which are applicable to a wide variety of tissue sources and species of origin.

Many techniques can lead to the production of an acellular collagen matrix or ECM. Gilbert, Sellaro and Badylak have elegantly reviewed the three method used for tissue decellularization: physical, chemical and enzymatic [49]. Physical methods include snap freezing that disrupt cells by forming ice crystals, high pressure that burst cells and agitation, that induce cell lysis and used most often in combination with chemical methods to facilitate penetration of active molecules in the tissue. Chemical methods of decellularization include a variety of reagents that can be use to remove the cellular content of ECM. These substances range from acid to alkaline treatments, as well as chelating agents such as EDTA, ionic or non-ionic detergents and solutions of extreme osmolarity. Enzymatic treatments such as trypsin, which specifically cleaves proteins and nucleases that remove DNA and RNA are also commonly used to produce acellular scaffold. However, none of these methods can produce an ECM completely free of cellular debris and a combination of techniques is often required to obtain a material free of any cell remnant.

The other type of collagen-based biomaterial is made by processing a collagen solution with other biomolecules like glycosaminoglycans (GAG) [50,51,52], elastin [53,54,55] or chitosan [56,57,58]. In order to produce collagen-based biomaterials, different approaches were developed to extract collagen from biological tissues. Modern extraction methods are based on three basic principles of solubilisation: in acid solutions [59,60], in neutral salt solutions [61,62] and in proteolytic solutions [63,64,65]. Proteolytic extraction however, alters collagen molecular structure by cleaving the terminal telopeptide regions and results in a proportional decrease in tropocollagen self-assembled fibrils [66]. To avoid this effect endogenous proteases can be inhibited during the acid solubilization [67]. Nonetheless, the acid extraction using a slight pepsin solubilization, is the most effective technique in terms of yield, albeit some telopeptides are cleaved or partially denaturated [67,68].

### 3.2. Crosslinking methods and reinforcement with biopolymers combination

Crosslinking techniques are especially important for collagen-based biomaterials when compared to acellular collagen matrices that have already been polymerized *in vivo*. As previously stated, the two types of collagen-based biomaterials can be crosslinked in order to enhance their mechanical and enzymatic resistance properties for implantation purposes. The principle of a cross-linking reaction relies on the modification of amine and carboxyl groups within the collagen molecules, to allow the formation of covalent bonds. Several methods have been developed to cross-link collagen scaffolds. These polymerization techniques are distributed among three types: physical, chemical and enzymatic crosslinking. 

Physical crosslinking rely on irradiation by ultra-violet wavelengths (UV) or thermal sources to induce the collagen scaffold polymerization. UV irradiation and dehydrothermal (DHT) treatment produce similar results when used to crosslink collagen scaffolds. Both techniques induce an increase in tensile strength and some fragmentation in the collagen molecular structure [69]. However, UV irradiation is more time-effective when compared to DHT treatment as it takes only 15 minutes instead of 3 to 5 days for the DHT treatment. UV crosslinked collagen scaffolds also result in a more suitable biomaterial for load-bearing applications due to its enhanced enzymatic resistance [13]. Besides, UV irradiation has been recently optimized to reduced collagen fragmentation by using glucose in the crosslinking process [70]. However, UV irradiation is only effective for thin and/or transparent scaffolds, allowing UV to go through the structure. 

The chemical techniques used to crosslink collagen-based biomaterial are more diversified. The use of aldehydes such as formaldehyde and glutaraldehyde was extensively used in the past decade. Glutaraldehyde is the most employed and studied chemical method used to crosslink collagen-based biomaterials [14,58,71,72]. Another class of chemicals used to enhance mechanical and enzymatic resistance of a collagen scaffold is the carbodiimide family [73,74,75,76]. These chemicals can also be used to crosslink collagen to some marginal substances like gold nanostructure [77] or utilized in combination with epoxy [78,79]. The isocyanate chemical family, especially hexamethylene diisocyanate, is also used to crosslink collagen scaffolds [80,81]. The commercially available product Zimmer® Collagen Repair Patch currently uses a proprietary isocyanate crosslinking technique. Genipin, a chemical cross-linker derived from a vegetal source, shows an interesting potential to replace glutaraldehyde because of its low toxicity [82,83]. However, all these chemical stabilisation techniques leave potentially toxic residues in the collagen-based biomaterial [84,85].

An alternative to covalent bond crosslinking is to promote the formation of ionic bonds between collagen molecules. This can be achieved by polycationic molecules such as chitosan, which create ionic bonds between its numerous amine groups and the carboxyl groups of collagen. These bonds are strong enough to stabilize the biomaterial structure and form a strong mechanical strength [57,86]. The major advantage of this technique is to prepare the biomaterial in a one step process, where chitosan is mixed with collagen before freeze-drying, avoiding the need of further washing steps since chitosan is not toxic [87].

Finally, enzymatic crosslinking agents like transglutaminase can be used to enhance tensile strength and enzymatic resistance of collagen-based biomaterial [55,88,89]. The major advantage with the approach of using a biologic polymerization technique is that no chemical residues or by-products remain in the scaffold structure, and therefore eliminate the risk of inducing cytotoxic effects.

A plethora of biomolecules can also be added to collagen solution to produce collagen-based biomaterials. These biomolecules, typically GAG, elastin and chitosan are added to the compound to potentially enhance the mechanical strength and to modulate cellular functions such as migration, proliferation and differentiation [90,91,92,93,94,95,96].

### 3.3. Sterilisation methods

The structure of collagen scaffolds, either crosslinked or decellularized, is relatively fragile and is temperature sensitive. Therefore, they are not autoclavable and require an alternate specific sterilization process prior to their use. Even if some sterilization of collagen-based biomaterials is done by low dose gamma irradiation (γ-ray), this method alters molecular structure and decreases mechanical and enzymatic resistance of the collagen scaffold [19,97,98,99]. The addition of glucose during irradiation has been investigated to increase sterilized scaffold tensile strength by forming glucose crosslinking, but does not avoid collagen structure degradation [100]. Ethylene oxide (ETO) sterilization or β-ray irradiation are less damageable than γ-ray but their applicability depends on the type of collagen-based biomaterials produced [86,101,102]. Electron beam irradiation, like γ-ray, induces a scaffold degradation that result in the lost of mechanical and enzymatic resistance [86,103]. Immersion in a low concentration of peracetic acid is the most commonly used method to sterilize acellular collagen ECM [104] and it has been demonstrated that formic acid can also be a potential sterilization agent for collagen [105]. Ethanol immersion with the combination of fungicide and antibiotic use are techniques used in the laboratory to sterilize collagen scaffolds which have been physically crosslinked [106,107]. Anyhow, no perfect sterilization technique have been recognized for use in collagen scaffolds without any molecular alteration to its structure. Investigating the sterilization effects on collagen material properties remains the best way to assess the performance of sterilized collagen-based biomaterials [108].

## 4. Recent Advances in Collagen-Based Biomaterials

### 4.1. Experimental applications

The use of collagen-based biomaterials, from either acellular matrix or extracted collagen, in fundamental studies have a vast range of applications both *in vivo* and *in vitro*. Research groups use collagen scaffolds to study cell behavior such as migration and proliferation, as well as differentiation and phenotype expression. Moreover, fundamental findings about how cells behave in complex environments rely on the capacity of cells to grow *in vitro* in a 3D tissue-like scaffold. Collagen hydrogels are also convenient scaffolds when the access to cell membrane is needed, for example in electrophysiological protocols [109,110,111]. Other collagen-based scaffolds are used as nervous system models to visualise motor neuron myelinisation by Schwann cells [112]. Cancer studies are also an important research topic where 3D collagen scaffolds are useful. In this way, the invasive character of cancer cells [113,114] and interaction between cancer cells and other cell types in a 3D environment can be analysed [115]. This kind of scaffold can also be used as a 3D environment to test anticancer drugs [116]. In the domain of immunology, *in vitro* 3D experiments can also be done to evaluate T cell migration mechanisms [117,118]. Furthermore, collagen-based biomaterials could serve as anchorage material to cultivate organs *ex vivo* [119] or as 3D models for diseases like osteoarthritis [120]. 

### 4.2. Osteochondral defects

Bone and cartilage reconstruction are important topics of modern medicine either for functional or esthetic surgery. Collagen-based biomaterial implantation is necessary when osteochondral defectS reach an important volume or when autograft have to be avoided for practical or pathological reasons. Scaffolds for bone tissue engineering rely on hardening of a collagen biomaterial by mineralization with calcium phosphate [121,122] and/or on crosslinking with other substances like hydroxyapatite [123,124,125] or bushite [126,127]. Collagen-based biomaterials used for cartilage regeneration tend to be more flexible and are ideally built with type II collagen in contrast to most of the other collagen-based biomaterials, which are produced using type I collagen. Nonetheless, some studies demonstrate that small amounts of autologous chondrocytes can grow in dynamic culture on type I or II collagen structures without any notable difference [128,129]. Sheet-like collagen scaffolds seeded with or without autologous cells can also be used to fill ostechondral defects [130,131,132,133]. Further developments aimed at differentiating mesenchymal stem cells directly in collagen-based biomaterial, to permanently solve osteochondral defects on a long-term basis, are currently under investigation [134]. Optimization of pore size and distribution is also a concern considering the effect of these parameters on cell adhesion, proliferation and migration [135]. Decellularization of complex structures like meniscus has also shown promising results in order to produce an optimal replacement scaffold for specific osteochondral defects [136,137]. 

### 4.3. Vascular diseases 

Two main problems arise in the domain of vascular diseases: cardiovascular malfunction and venous or arterial pathologies such as atherosclerosis. In the case of heart diseases, tissue engineering solutions rely principally on acellular matrix colonization and implantation due to the complex structural architecture of the heart like heart valves [138,139]. However, the usefulness of xenogenic acellular heart valves remain an issue due to their important immunogenic potential and tendency to become calcified [140]. This immunologic issue led to the development of commercially available human acellular scaffolds for cardiac and vascular reconstructive surgeries, *i.e.* Cryolife^®^ that offers tissue replacement ranging from heart valves to vascular conduits [141,142,143]. Recent findings about complete heart decellularization by perfusion [144] and production of functional re-endothelialized veins and valves from human vein matrix [145] will certainly lead to the development of very important advances in cardiovascular regenerative medicine using collagen-based biomaterials.

A very innovative personalized medicine approach, to reconstruct living tissue-enginneered blood vessels using the patient’s own cells, has been developed at the LOEX by the group of Auger *et al.* [146]. The idea is to let human fibroblasts produce their own collagen-made extracellular matrix through long-term culture. This self-assembled fibroblast sheet can then be rolled around a tubular support to produce a living vessel with very impressive mechanical strength and biological properties after *in vitro* maturation [146,147,148]. This tissue-engineered blood vessel has been successfully grafted on patients [149,150].

### 4.4. Skin and cornea

Skin and cornea share a similar tissue structure: dermis and stroma both being connective tissues; epidermis and cornea being stratified epithelia. Collagen-based wound dressings have been applied for decades for burn coverage applications and ulcer treatment [28,151,152]. Highly sophisticated and innovative tissue-engineered skin models have been developed with melanocytes [153], a capillary-like network [154], dendritic cells [155], sensory innervation [94,156], adipose tissue [157], and tissue reproducing psoriatic or sclerotic phenotypes [158,159]. A living allogenic reconstructed skin (Apligraf^®^), made of a collagen gel-populated fibroblasts overlayed by an epidermis, is commercialized for ulcer treatment as a temporary dressing [160,161]. Skin, dermal substitutes and dressing such as Integra^®^ (acellular collagen-GAG scaffold), Alloderm^™^ (human dermis), Amniograph^™^ (amniotic membrane) and Oasis^™^ (porcine SIS) are currently available for medical applications. Mesenchymal stem cell delivery to the wound bed in collagen-based biomaterial is a growing topic in wound healing [157,162,163,164]. The combination of collagenous biomaterials and stem cells could also be a valuable strategy to treat corneal defects. In the last decade, collagen scaffolds have been intensively studied for the delivery of limbal epithelial stem cells to damaged cornea [165,166,167,168,169,170]. Advances in collagen-based corneal scaffolds also include the utilization of recombinant human collagen [169,171,172,173], the secretion of collagen by the fibroblasts themselves (self-assembled fibroblasts sheets) [174] and surface modification to reduce extensive endothelialization [175].

### 4.5. Urogenital system

The use of collagen-based biomaterials in the domain of urogenital diseases and dysfunctions rely principally on acellular ECM from either SIS or BSM. These scaffolds have been extensively used to replace the enterocystoplasty and gastrocystoplasty techniques that were previously used with frequent complications [176,177]. Hence, more recent surgical procedures aiming to solve genitourinary disorders use acellular collagen scaffolds in bladder augmentation [178,179,180,181] and urethral stricture [182,183,184]. Although acellular matrix is currently evolving and slowly becoming the new gold standard for these surgeries, collagen-composite scaffolds populated with the patient’s own urothelial and muscle cells or self-assembled fibroblast sheets are also a promising strategy for bladder augmentation [185,186,187] and are showing optimistic clinical results [186]. Vesico-urethral reflux and incontinence are other defects of the urogenital system which can also be solved using injection of collagen biomaterials [188,189,190].

### 4.6. Neural migration 

Peripheral nerve regeneration is a very important topic in regenerative medicine. Collagen-based biomaterials have been extensively studied as a promising nerve guide [191,192,193]. Multiple compositions of collagen-based nerve conduits have recently been tested with positive results compared to clinically used autografts. Even if acellular scaffolds have shown good results [194], most collagen nerve guides are engineered from crosslinked collagen solution molded into tubular shape like commercially available NeuraGen^®^ from Integra^™^. Pore orientation [195,196], addition of neurotrophic factors [197,198] and cell delivery [199,200,201] are currently being investigated in an attempt to enhanced nerve guides for clinical applications. Collagen-based biomaterials can also be used to develop innovative three-dimensional tissue-engineered nervous system models to promote 3D axonal migration and myelination of sensory or motor neurons by Schwann cells through a connective tissue [107,112,202].

### 4.7. Dermal filler, wound dressing and delivery systems 

FDA approved dermal filler commonly used in facial rejuvenation or reconstructive surgery is using collagen from three distinct sources: Bovine Zyderm^®^, porcine Evolence™, human CosmoDerm^®^ and Cymetra^®^ [203,204]. Although other collagen-based biomaterials are available for this purpose [205], these products can be useful for medical office-based interventions. The overgrowing popularity for these collagen-based dermal fillers is also due to the long-term side effect observed with non-degradable fillers like Bio-Alcamid^™^, which are susceptible to form granulomas [206,207]. 

Wound dressings that are also delivery systems represent an interesting application for collagen-based applications. Recent studies have shown the feasibility and more importantly the benefits of implants delivering antibiotics [208,209,210,211]. The delivery properties of collagen-based biomaterials also display great potential for ulcer treatment [197] and abdominal wall defect reconstruction [212,213,214]. Collagen scaffolds have also shown to accurately deliver cells, proteins, drugs and nucleic acids on a predictable and long-term basis [129,215,216,217]. Finally, a recent clinical trial using adenovirus in collagen gel has cleared the path for future clinical studies on gene therapy delivered by collagen matrix [218]. The biodegradability of collagen and its low immunogenicity make it a substrate of choice for internal and topical pharmacogenomical applications. 

## 5. Conclusion

Collagen-based biomaterials are of the utmost importance for tissue engineering and regenerative medicine. Because of its superior biocompatibility and low immunogenicity, collagen is still the protein of choice for biomaterials preparation. It can be extracted from various tissue sources and assembled in combination with other molecules. There is also a use in the laboratory as a decellularized ECM in fundamental studies or as tissue replacement material in medical applications. Most present research is aimed at the optimization of collagen-based biomaterials for medical applications by enhancing mechanical strength, biodegradability or delivery characteristics. 
